# Attitudes of medical students to medical leadership and management: a systematic review to inform curriculum development

**DOI:** 10.1186/1472-6920-11-93

**Published:** 2011-11-14

**Authors:** Mark R Abbas, Thelma A Quince, Diana F Wood, John A Benson

**Affiliations:** 1 General Practice and Primary Care Research Unit, University of Cambridge, Forvie Site, Robinson way, Cambridge, CB2 0SR, UK; 2 University of Cambridge School of Clinical Medicine, Box 111 Addenbrookes Hospital, Hills Road, Cambridge CB2 2SP, UK

## Abstract

**Background:**

There is a growing acknowledgement that doctors need to develop leadership and management competences to become more actively involved in the planning, delivery and transformation of patient services. We undertook a systematic review of what is known concerning the knowledge, skills and attitudes of medical students regarding leadership and management. Here we report the results pertaining to the attitudes of students to provide evidence to inform curriculum development in this developing field of medical education.

**Methods:**

We searched major electronic databases and citation indexes within the disciplines of medicine, education, social science and management. We undertook hand searching of major journals, and reference and citation tracking. We accessed websites of UK medical institutions and contacted individuals working within the field.

**Results:**

26 studies were included. Most were conducted in the USA, using mainly quantitative methods. We used inductive analysis of the topics addressed by each study to identity five main content areas: Quality Improvement; Managed Care, Use of Resources and Costs; General Leadership and Management; Role of the Doctor, and Patient Safety. Students have positive attitudes to clinical practice guidelines, quality improvement techniques and multidisciplinary teamwork, but mixed attitudes to managed care, cost containment and medical error. Education interventions had variable effects on students' attitudes. Medical students perceive a need for leadership and management education but identified lack of curriculum time and disinterest in some activities as potential barriers to implementation.

**Conclusions:**

The findings from our review may reflect the relatively little emphasis given to leadership and management in medical curricula. However, students recognise a need to develop leadership and management competences. Although further work needs to be undertaken, using rigorous methods, to identify the most effective and cost-effective curriculum innovations, this review offers the only currently available summary of work examining the attitudes of students to this important area of development for future doctors.

## Background

There is a growing acknowledgement that doctors need to develop leadership and management competences. The Department of Health white paper 'Equity and excellence: Liberating the NHS' (2010) aims to empower health professionals by giving more control and decision-making to frontline staff [1]. In Tomorrow's Doctors (2009), the General Medical Council includes competences relating to effective multi-professional team working, ability to protect patients and improve care [2].

The NHS Institute for Innovation and Improvement and Academy of Medical Royal Colleges have developed the Medical Leadership Competency Framework (MLCF) [3], which describes the leadership competences that doctors need, in order to become more actively involved in the planning, delivery and transformation of patient services. They have also produced a curriculum guide intended for use by UK medical schools [4]. When developing curricula to meet the challenges raised by the need to prepare students for responsibilities in management and leadership, it will be necessary to recognise the attitudes of students themselves, since these are likely to influence students' engagement with planned educational activity. We undertook a systematic review of what is known concerning the knowledge, skills and attitudes of medical students regarding leadership and management. Here we report the results pertaining to the attitudes of students to provide evidence to inform curriculum development in this developing field of medical education and to support practising clinicians considering how to aid the development of students into the doctors required by a changing NHS. The review addressed the following two questions:

1. What are medical students' attitudes towards medical leadership and managers/management in the health sector?

2. What are medical students' attitudes towards medical leadership and management education and training?

## Methods

### Ethical approval

Ethical approval was not required for this study.

### Search strategy

There are no concensus definitions in the literature for leadership and management; indeed, there is debate about the differentiation between the two terms. For the purpose of this review, we defined medical leadership and management as work which addressed strategy, delivery and innovation within medical organizations. We included reports available in English of any empirical work addressing either of the review questions. There was no date restriction. We followed the following steps:

#### Electronic Databases Search

Between March and July 2009, we searched major electronic databases and citation indexes within the disciplines of medicine, education, social science and management. Searches for Pubmed, Embase, Web of Knowledge and ERIC are provided (Additional File 1). We also searched BREI, AUEI, Scopus, Healthcare Business Fulltext Elite, HMIC and JSTOR but no additional citations were found. Due to the difficulty in differentiating management related to organisations and management related to patient care, we used a very inclusive search strategy which yielded a large number of titles.

#### Title Assessment Phase

Two reviewers (MA/JB) screened titles for relevance against the stated literature review aims. We sought to obtain the abstracts of titles selected by either reviewer. If the abstract was unavailable, we sought the full paper. *Abstract Assessment Phase: *Two reviewers (MA/TQ) assessed the abstracts for relevance against the review aims. We sought to obtain the full papers of selected abstracts. *Full Paper Assessment Phase: *Two reviewers (MA/TQ) assessed the full papers for relevance (as before). JB moderated in the case of disagreement. *Hand searching of major journals: *One reviewer (MA) searched major journals in the medical education and management literatures (Medical Teacher, Medical Education, Clinician in Management, Journal of Health Services Research and Policy, Journal of Health Organisation and Management (formerly Journal of Management in Medicine), Health Services Management Research). Using the methodological phases described above, relevant papers went forward to the Quality Assessment Phase.

#### Snowballing

Two reviewers (MA/TQ) undertook reference tracking and citation tracking for papers included in the final review. Using the methodological phases described above, relevant papers went forward to the Quality Assessment Phase.

#### Accessing of grey literature

One reviewer (MA) accessed websites of UK medical institutions and attempted to contact individuals working within the field with a request to supply relevant documentation. No relevant papers were identified.

### Quality Assessment Phase

Two reviewers (MA/TQ) undertook a global evaluation of each full paper. Evaluation included: assessment of overall quality of the study in its own terms; appropriateness of the form of evidence, and relevance of the study findings in answering the review question. Papers were categorised according to 5 "global" ratings: low, low/moderate, moderate, moderate/high and high. Papers evaluated as 'low' were excluded from the Data Extraction Phase. All other papers went forward to the Data Extraction Phase.

### Data Extraction Phase

The data extraction form was developed by all three reviewers, informed by other data extraction protocols (e.g. EPPI-Centre (2007)) but modified to include important aspects specific to this review. Two reviewers (MA/TQ) extracted data regarding: context of study, recruitment, description of participants, study design, results and conclusions. JB moderated in the case of disagreement.

## Results

### Characteristics of Eligible Studies

**Figure 1:**
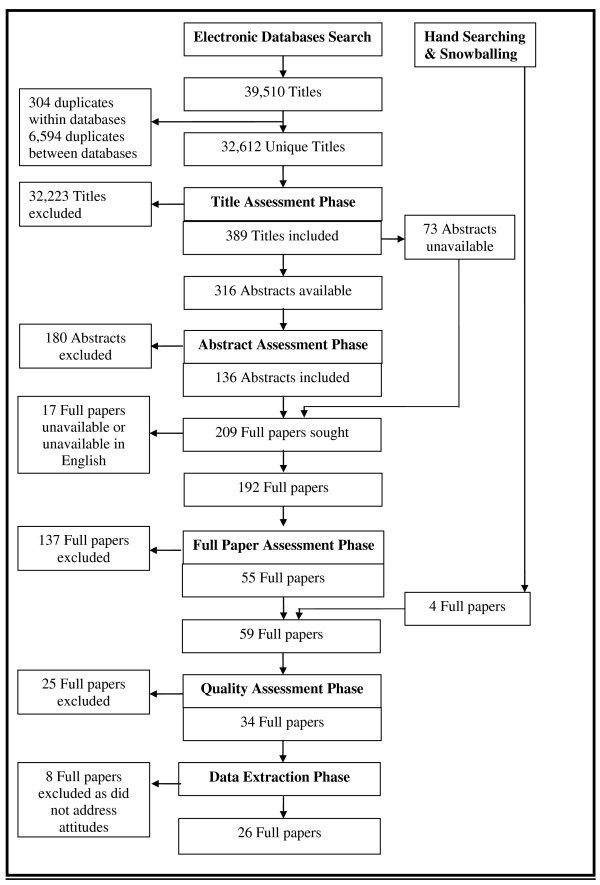
**Flowchart for paper inclusion in the review**.

26 studies were included in the review (Figure 1: Flowchart for paper inclusion in the review). Table 1 summarises the characteristics of included studies. Most had been conducted in the US and employed mainly quantitative methods.

**Table 1:** Characteristics of Studies Included in the Review (n = 26)

Date of Publication		National Setting		Methodology	
**Date**	**No.**	**Country**	**No.**	**Methodology**	**No.**

Before 1980	0	US	18	Quantitative only	13
1980-1989	2	UK	6	Qualitative only	1
1990-1999	7	Other	3	Mixed	12
2000-March 2009	17	Portugal (1)			
		Israel (1)			
		Sweden (1)			

Note: Martins *et al *(2005) included in both UK and Other-Portugal

We used inductive analysis of the topics addressed by each study to identity five main content areas, related to the review's two questions about medical students: attitudes to leadership and management (ALM) and attitudes to education and training for leadership and management (AET). (Table 2).

**Table 2:** Number of studies addressing each content area and study question

Content Areas	Review questions		
	ALM	AET	
Quality Improvement	2	4	
Managed Care, use of Resources and Costs	6	6	
General Leadership and Management	3	3	
Role of the Doctor	3	0	
Patient Safety	2	2	

Total^1^	16	15	

Note*^1^*: number of papers sum to greater than 26 because some papers covered multiple content

Reporting of individual studies is restricted to only those aspects pertaining to leadership and management. A detailed summary of the outcome measures and results of included studies within the five content areas is provided in Additional File 2: Summary of Included Studies.

### Quality Improvement

Four studies explored student attitudes relating to quality improvement including medical audit [5-8].

#### Attitudes of medical students toward leadership and management

McCurdy *et al *(2003) described the responses of students to a national graduation survey [5]. Students agreed with the use of clinical practice guidelines and need for cost containment but no differences were found in attitudes between students who completed a patient care report and a control group. Another study, assessing attitudes towards diabetes quality improvement projects and qualitative content analysis, revealed that students recognised the importance of well organised charts and potential benefits of quality improvement techniques in improving care [6].

#### Attitudes of medical students toward leadership and management education

All four studies in this section evaluated an education intervention [5-8]. Results were mixed. Henley (2002) found that students were generally positive towards the activity, as a learning experience and its ability to influence patient care [7]. However, Gould *et a*l (2002) and Morrison & Sullivan (1993) found that while recognising audit's relevance, students reported frustration with the activity itself and were concerned about lack of project support and efficiency of projects given competing educational demands [6,8]. McCurdy *et al *(2003) found no difference between intervention and control groups for 5 items on the UME 21 Graduation Survey which assessed exposure to aspects of managing patient care [5]. None of the studies presented data confirming the psychometric properties of the instruments used to assess attitudes.

### Managed care, Use of resources and Costs

Twelve studies explored aspects in this area [9-20].

#### Attitudes of medical students toward leadership and management

Six studies explored medical students' attitudes towards principles of managed care and cost containment [9-14]. Students' attitudes were mixed. Medical students appeared to accept the necessity for managed care and cost-containment, and perceived a need to adapt to managed care [9]. However, other studies revealed students were concerned about possible negative effects on the quality of care, the doctor-patient relationship [10] and physicians' independence [11]. In a US study male students, interested in prestige and opposed to healthcare rationing, were more likely to have negative attitudes towards managed care than students interested in improving access to to care and having a personal interest in primary care careers [11,12].

Three studies involved an education intervention. Lazarus *et al *(1998) and O'Connell *et al *(2004) found positive changes in attitudes post-intervention [9,13] while Williams *et al *(1984) found no change in attitudes towards managed care following a cost containment educational programme [14]. O'Connell *et al *(2004) examined medical students' attitudes and knowledge about Managed Care Organisations and assessed the effects of a teaching programme that spanned 4 years [13]. Positive changes were found only immediately after a visit to the administrative offices of a non-profit Managed Care Organisation. Only Lazarus *et al *(1998) used validated instruments to assess attitudes [9].

#### Attitudes of medical students toward leadership and management education

Six studies explored medical students' attitudes towards education about managed care and use of non-human resources [15-20]. Five studies examined students' perceptions of the adequacy of their medical curricula [15-19]. Dissatisfaction was reported in content areas addressing: practice management, risk management, utilization review and quality assurance, cost effective medical practice, medical care costs and cost control, medical social economics, health policy, healthcare delivery and health care reform issues.

One study involved an education intervention in the form of an evaluation of a community resource allocation project involving an ongoing service problem [20]. This was rated positively by most students.

### General leadership and management

Four studies explored medical students' attitudes regarding general leadership and management [21-24].

#### Attitudes of medical students toward leadership and management

Two studies found the majority of students considered general leadership and management skills important [21,22]. The skills considered more important were communication skills, ethics, conflict resolution, time management, managed care, management principles, coding and billing, quality improvement, public speaking and risk management. Skills considered less important include negotiating, writing proposals and investment.

Carufel Wert (2007) evaluated an education intervention: students valued highly 'Collaborating/negotiating with others involved in the implementation of my project', and there was some positive impact on 'Interest in taking leadership positions' but the highest impact was on 'Commitment to community services as a physician' [23]. No studies used validated instruments to assess attitudes.

#### Attitudes of medical students toward leadership and management education

Three studies addressed medical students' attitudes towards leadership and management education generally [21,23,24].

In a survey of University of Cambridge and New University of Lisbon students, Martins *et al *(2005) found approximately half thought a management course would be relevant, with Lisbon students more positive than Cambridge students [24]. Reasons cited for high relevance included "Improvement of self-confidence", "Increase NHS efficiency/effectiveness", "Necessary role of doctors", and "Ability to assume further responsibilities". However, curriculum pressures and the view that doctors should be mainly concerned with patients were given as justifications for low relevance.

In the above study most students considered that a leadership/management course should last one semester during the clinical years [24]. In conjunction with another study, students felt that any course should cover: leadership, team management, communication and resource management [21,24]. The desire for leadership training and experience was a common reason for enrolling in a community oriented quality improvement programme [23]. None of the studies used validated instruments to assess attitudes.

### Role of Doctor

Three studies addressed medical students' attitudes towards the health care team and role of the doctor [25-27]. None of the three studies assessed attitudes to an educational intervention and none used validated instruments.

Two studies found medical students had generally positive attitudes to the multidisciplinary team and appreciated their influence on patient care [25,26]. Harward (2006) found students believed teamwork improves patient care quality and holistic care, fosters enthusiasm, and considered 'collaborative care planning', 'understanding others' terminology' and 'negotiating treatment and follow up' important [25]. An interdisciplinary case conference augmented these beliefs. Pre-intervention, students held relatively high positive views of the importance of care provided by other health professionals except audiologists, speech pathologists and rehabilitiation counsellors. However students in two studies believed that doctors should lead the team [25,26], even though in one of these studies students rejected hierarchial organization. One study found students' attitude scores were only midrange towards doctors having managerial roles in trusts and personal interests in management [27].

### Patient Safety

Three studies addressed medical students' skills, knowledge of and attitudes towards patient safety [28-30]. None of the studies used validated instruments.

#### Attitudes of medical students toward leadership and management

Two studies explored students' attitudes to aspects of patient safety [28,29]. Both studies focussed on aspects that students could affect, and results were mixed. Both studies revealed students' reluctance to report errors and criticism toward role modelling displayed by doctors and faculty.

Both studies included education interventions, which showed mixed or negative effects on student attitudes [28,29]. Goldie *et al *(2003) found student responses to a vignette addressing whistleblowing frequently reflected personal attitudes or values rather than professional consensus [28].

#### Attitudes of medical students toward leadership and management education

Two studies explored students' attitudes to patient safety curricula [29,30]. Students expressed positive attitudes towards the interventions and reported gaining confidence in managing medical errors [29,30].

## Discussion

To the authors' knowledge, this is the first systematic review exploring the attitudes of undergraduate medical students with regard to leadership and management.

Our review indicates that students agreed with the use of audit, clinical practice guidelines and quality improvement techniques. Students had mixed attitudes to the principles of managed care, use of resources and costs It is important to acknowledge the term 'managed care' may have different meanings between studies across different healthcare systems, which may subsequently have affected student attitudes. Most included studies were conducted in the USA, where managed care has been employed much more widely since the implementation of the Health Maintenance Organization Act (1973), with the explicit emphasis on reduction of healthcare costs as well as improvement of healthcare quality. Furthermore, student attitudes may reflect the currently peripheral focus given to leadership and management within medical education.

The mixed findings with regard to patient safety, especially reluctance of students to report medical error is disappointing and is in direct conflict with much guidance. For example, in the UK, Tomorrow's Doctors (2009) states how 'students must appreciate the importance of protecting patients, even if this conflicts with their own interests or those of friends or colleagues. If students have concerns about patient safety, they must report these to their medical school' [31]. Therefore faculties of medical education will have to encourage the development of student attitudes in this area. In addition to the responsibility of medical students, Tomorrow's Doctors (2009) highlights the responsibilities also of the medical school to have systems and procedures that 'inform students, and those delivering medical education, of their responsibility to raise concerns if they identify risks to patient safety, and provide ways to do this... Medical schools must provide robust ways for concerns to be reported in confidence and communicate these to students' [32]. Indeed this could be expanded further to emphasise the importance of remedial action where applicable by liaison with healthcare providers to improve the standards and processes of care. Furthermore, students would ideally be enabled to take an active role in this process.

The finding that medical students generally have positive attitudes towards multidisciplinary teams but believe that doctors lead or should lead the team may be seen positively or negatively. Students seem prepared to take on leadership roles within the clinical team, but may be less interested in being followers within teams or leading on management issues. These findings are important to curriculum planners and clinicians preparing students for a workplace in which a model of distributed leadership is encouraged.

The effects of educational interventions on attitudes to management and leadership were variable. This finding is in keeping with previous research on the effect of educational interventions on attitudes generally. Students were positive toward many aspects of leadership and management. They also perceived a need for leadership and management education in the undergraduate medical curriculum. However, possible barriers to implementation within medical schools are lack of time given competing educational demands, and possible disinterest in the activity itself on the part of some students and.faculty. Most education interventions described in this review occurred during clinical training and students may prefer any leadership and management education in this context. Curriculum planners will therefore need to be flexible if they are to successfully incorporate leadership and management training. It may be more effective and efficient to help students relate ongoing educational activities with relevant leadership and management education rather than running an isolated leadership and management course. In this regard, it may first be necessary to win the hearts and minds of clinicians with whom students come into contact in order that they can act as positive role models for the importance of leadership and management in improving patient care and assist students develop their competences as they develop their clinical expertise.

Due to the difficulty in differentiating management related to organisations and management related to patient care, we used a very inclusive search strategy, which yielded a large number of titles. However this strategy also resulted in consideration of material not specifically generated for healthcare audiences. The healthcare sector may benefit from looking at other sectors. In a review of published quality improvement curricula for clinicians, Boonyasai *et al *(2007) also identified a large number of potentially relevant citations and two authors undertook a title assessment phase, similar to our study [33]. In this review, few actual studies pertaining to leadership and management were identified. Most of the studies included provided minimal demographic information about participants who were largely self-selected. Most studies employed mainly quantitative methodologies but used unvalidated instruments to examine predominantly short term outcomes. Most studies were undertaken in the USA, which has a very different health care system from the UK and much of Europe. Most significantly, managed care has been employed much more widely in the USA, since the implementation of the Health Maintenance Organization Act (1973), with the explicit emphasis on reduction of healthcare costs as well as improvement of healthcare quality. Caution is therefore needed in interpreting the generalisability of these results, and the significance of changes resulting from educational interventions.

## Conclusions

Medical students have mixed attitudes to aspects of leadership and management, and education interventions had variable effects on students' attitudes. Although further work needs to be undertaken, using rigorous methods, to identify the most effective and cost-effective curriculum innovations to enable medical students to develop relevant skills to achieve service delivery and improvement, there is a growing acknowledgement that doctors need increasingly to develop leadership and management competences. This review offers the only currently available summary of work examining the attitudes of students to this important area of development for future doctors.

## Competing interests

Mark Abbas and John Benson have collaborated with the NHS Institute for Innovation and Improvement to produce an undergraduate curriculum guidance document addressing medical leadership. Reference: NHS Institute for Innovation and Improvement and Academy of Medical Royal Colleges. Guidance for Undergraduate Medical Education: Integrating the Medical Leadership Competency Framework. Coventry, 2010.

Diana Wood has collaborated with the NHS Institute for Innovation and Improvement as a steering group committee member of the Enhancing Engagement in Medical Leadership Project.

None of the authors has any financial or other competing interests.

## Authors' contributions

MRA provided initial concept for area of study of leadership and management. MRA, TAQ and JAB contributed to the further development and design of the study. MRA, TAQ. and JAB contributed to data acquisition and analysis as described in the methods section. M.R.A. wrote the paper with substantial contributions from TAQ and JAB. DFW provided advice regarding final draft submission. All four authors contributed to the final draft submission.

All four authors have given final approval of the version to be published. M.R.A. is the guarantor of the final version to be published

## Pre-publication history

The pre-publication history for this paper can be accessed here:

http://www.biomedcentral.com/1472-6920/11/93/prepub

## Supplementary Material

Additional file 1 **Search strategies**. Searches for Pubmed, Embase, Web of Knowledge and ERIC. Click here for file

Additional file 2 **Summary of included studies**. Detailed summary of the outcome measures and results of included studies within the five content areas. Click here for file
