# Supplementary material for: Attitudes of medical students to medical leadership and management: a systematic review to inform curriculum development
Source: BMC Med Educ. 2011 Nov 14;11:93. doi: 10.1186/1472-6920-11-93 (PMC3247079; doi:10.1186/1472-6920-11-93)
Supplement: Additional file 1 — Search strategies. Searches for Pubmed, Embase, Web of Knowledge and ERIC. [file 1472-6920-11-93-S1.DOC]

**Additional File 1: Search Strategies**

Searches for Pubmed, Embase, Web of Knowledge and ERIC are provided. We also searched Scopus and Healthcare Business Fulltext Elite, HMIC and JSTOR (derived from the Pubmed search strategy) and BREI and AUEI (derived from the ERIC search strategy). The additional search strategies are available on request.

*Pubmed Search Strategy*

((((("Students, Medical"[Mesh] OR "Education, Medical, Undergraduate"[Mesh])) OR (((((((((("medical student")) OR (("medical students"))) OR (("premedical student"))) OR (("premedical students"))) OR (("clinical student"))) OR (("clinical students"))) OR (("student doctor"))) OR (("student doctors")))))) AND ((((lead OR leader OR leaders OR leadership OR manage OR managing OR manager OR managers OR management OR organisation OR organisations OR organisational OR organization OR organizations OR organizational)) OR (("Health Care Category"[Mesh] OR "Leadership"[Mesh]))))) AND (((((((((attitude OR attitudes OR attitudinal OR skill OR skills OR know OR knowledge OR behave OR behaviour OR behaviours OR behavior OR behaviors)) OR (("Knowledge"[Mesh]))) OR (("Social Behavior"[Mesh]))) OR (("Communication"[Mesh]))) OR (("Achievement"[Mesh]))) OR (("Aptitude"[Mesh]))) OR (("Attitude"[Mesh] NOT ("Attitude to Computers"[Mesh] OR "Attitude to Death"[Mesh])))))

*Embase*

1. medical students.mp. or exp Medical Student/

2. exp economic aspect/ or exp management/ or “social aspects and related phenomena”/ or exp “health care facilities and services”/ or exp health care personnel/ or exp health care management/ or exp health economics/ or exp health care organization/ or exp health care quality/ or exp disease management/ or exp economics/ or exp finance/

3. know$.mp. or exp knowledge/ or exp nursing knowledge/ or exp professional knowledge/ or skill.mp. or exp Skill/ or exp Communication Skill/ or exp Skill Retention/ or exp Skill Mix or attitude$.mp. or exp attitude/ or exp attitude to change/ or attitude to health/ exp consumer attitude/ or exp cultural bias/ or exp cultural sensitivity/ or exp employee attitude/ or exp student attitude/ or behave$.mp. or exp Behavior/

4. 1 and 2 and 3

*Web of Knowledge*

# 1 TS=((medic* or premedical* or clinical or doctor*) SAME stud*) OR TS=((medic* or clinical) SAME (school* or educat*))

*Databases=SCI-EXPANDED, SSCI, A&HCI, CPCI-S Timespan=1900-2009*

# 2 TS=(manag* or lead* or organisa* or organiza*)

*Databases=SCI-EXPANDED, SSCI, A&HCI, CPCI-S Timespan=1900-2009*

# 3 TS=(know* or skill* or attitud* or aptitud* or achiev* or behav*)

*Databases=SCI-EXPANDED, SSCI, A&HCI, CPCI-S Timespan=1900-2009*

# 4 #1 and #2 and #3

*Databases=SCI-EXPANDED, SSCI, A&HCI, CPCI-S Timespan=1900-2009*

*ERIC*

1. MEDICAL-EDUCATION#.DE. OR MEDICAL-STUDENTS#.DE.

2. (MEDIC$3 OR PREMEDICAL$ OR CLINICAL OR DOCTOR$) NEAR STUD$4

3. 1 OR 2

4. manag$5 OR lead$6

5. organisa$ OR organiza$

6. Governance#.W..DE.

7. Organization#.W..DE.

8. Cost-Effectiveness#.DE. OR Costs#.W..DE.

9. Planning#.W..DE.

10. Change-Management#.DE. OR Contingency-Management#.DE. OR Participative-Decision-Making#.DE. OR Management-Development#.DE. OR Administrator-Education#.DE. OR Management-Information-Systems#.DE. OR Personnel-Management#.DE. OR Management-Systems#.DE. OR Management-Teams#.DE. OR Management-Development#.DE.

11. Leadership#.W..DE.

12. Leadership-Qualities#.DE. OR Leadership-Responsibility#.DE. OR Student-Leadership#.DE. OR Leadership-Styles#.DE. OR Leadership-Training#.DE. OR BUSINESS-ADMINISTRATION-EDUCATION#.DE.

13. 4 OR 5 OR 6 OR 7 OR 8 OR 9 OR 10 OR 11 OR 12

14. know$5 OR skill$ OR attitud$ OR behav$

15. Achievement#.W..DE. OR Ability#.W..DE.

16. Knowledge-Base-For-Teaching#.DE. OR Knowledge-Level#.DE. OR Metacognition#.W..DE. OR Feedback#.W..DE. OR Prior-Learning#.DE. OR Self-Concept#.DE.

17. Attitude-Change#.DE. OR Attitude-Measures#.DE. OR Attitudes#.W..DE. OR Work-Attitudes#.DE.

18. Student-Behavior#.DE.

19. Self-Control#.DE.

20. 14 OR 15 OR 16 OR 17 OR 18 OR 19

21. 3 AND 13 AND 20
