# Supplementary material for: Attitudes of medical students to medical leadership and management: a systematic review to inform curriculum development
Source: BMC Med Educ. 2011 Nov 14;11:93. doi: 10.1186/1472-6920-11-93 (PMC3247079; doi:10.1186/1472-6920-11-93)
Supplement: Additional file 2 — Summary of included studies. Detailed summary of the outcome measures and results of included studies within the five content areas. [file 1472-6920-11-93-S2.DOC]

**Additional File 2: Summary of Included Studies**

**Content Area: Quality Improvement (QI)**

| **Gould et. al. (2002)**  ***Aims:*** Examination of impact of a QI curriculum on educational outcomes and effects of student projects on quality of care.  ***Setting:*** US, primary care, 77 2^nd^ year medical students: audit of Type II diabetes care.  ***Design:*** Mixed methods. Quantitative pre and post training questionnaire and assessment of activity on both process and outcome indicators at the practice level pre and post intervention. Qualitative methods for attitudinal assessment of activity. | | |
| --- | --- | --- |
|  | **Outcome measures** | **Results** |
| **ALM** | Attitudes towards QI assessed by post intervention evaluation survey: frequencies and examination of qualitative content. | Post intervention evaluation survey frequencies: 43% reported appreciation of QI (32% neutral), 45% agreed chart audit beneficial to office practice: 35% felt beneficial to patient (31% felt not of benefit).  Qualitative content analysis: Students recognised importance of well organised charts and potential benefits of QI in improving care. |
| **AET** | Attitudes towards the QI project assessed by post intervention evaluation survey: frequencies and examination of qualitative content. | Post intervention evaluation survey frequencies: 42% did not consider project a valuable learning experience (43% neutral).  Qualitative content analysis: reported frustration with the activity itself and concern about time taken for the project. |
| **Henley (2002)**  ***Aims:*** Evaluation of knowledge, skills and attitudes in medical students undertaking QI module.  ***Setting:*** US, primary care, 30 3^rd^ year medical students: audit of Type II diabetes care.  ***Design:*** Mixed methods. Quantitative: skill assessed by completion of project and ability to develop improvement recommendations; knowledge assessed by 6 question non-graded quiz on completion of project; Attitudes assessed by 5 point Likert scale survey on completion of project. Qualitative- focus group interviews with 3 QI coordinators. | | |
|  | **Outcome measures** | **Results** |
| **AET** | Distribution of levels of satisfaction with module revealed by evaluation survey.  Themes generated by focus group interviews with QI co-ordinators | Levels of satisfaction with module: 71 % agreed performing audits was useful (0% disagreed), 50% agreed that developing an improvement plan was a practical learning experience (17% disagreed), 50% agreed that project provided opportunity to influence care (8% disagreed) and 71% felt that it was an appropriate activity at this stage in their course (8% disagreed).  Focus group themes: Students more receptive to the idea of QI when presented as important in improving patient outcomes and satisfaction. |
| **Morrison & Sullivan (1993)**  ***Aims:*** Evaluation of the first year of QI exercise addressing audit.  ***Setting:*** UK, primary care, 153 final (4^th^) year medical students: audit of Type II diabetes care.  ***Design:*** Mixed methods. Quantitative and qualitative evaluation of completed data collection forms; post intervention likert scales assessment of attitudes to intervention and knowledge of audit. | | |
|  | **Outcome measures** | **Results** |
| **AET** | Students’ levels of interest post intervention.  Relevance of audit exercise and feedback sessions.  Assessment of suitability of audit as topic for inclusion in course. | Audit exercise: 30% found “interesting”, 39% “boring”, 57% found “relevant”, 21% “poorly relevant or irrelevant”.  Feedback sessions: 60% found “interesting” 15% considered “boring” and 69% found “relevant” 8%, “poorly relevant or irrelevant”.  Suitability of audit for inclusion in course: 63.4% considered suitable. Overall attitude: 50% of students did not consider project to have been a waste of time (15% thought it a waste of time.) |
| **McCurdy et. al. (2003)**  ***Aims****:* Evaluation of student Patient Care Project (PCP) to develop understanding of a] discharge summary report b] QI methodology c] evidence based medicine d] cost awareness.  ***Setting:***US, hospital based, 239 final (4^th^) year medical students. Student selected subject.  ***Design:*** Mixed methods. Qualitative analysis of Patient Care Project reports. Quantitative: students completed anonymously administered quantitative national questionnaire (UME-21 Graduation Questionnaire) with control group of students who graduated in 1999. | | |
|  | **Outcome measures** | **Results** |
| **ALM** | Comparison of scores for 2 items of UME 21 Graduation survey assessing attitudes towards clinical practice management. | Most students undertaking PCP agreed with use of clinical practice guidelines: mean scores varied from 3.52 (SD 1.0) to 4.03 (SD 0.86) and need for cost containment (mean scores varied from 3.86 (SD .79) to 4.00 (SD.89). No statistically significant differences between intervention and control groups. |
| **AET** | Comparison of scores for 5 items of UME 21 Graduation survey assessing exposure to specific aspects of managing patient care covered by PCP: identification of costs, design of QI loop, use of evidence from Cochrane or similar, use of internet to access medical information and use of formal practice guidelines. | No statistically significant differences between intervention and control group in extent to which they considered they had undertaken these activities. |

**Content Area: Managed care, use of resources and costs**

| **Williams et. al. (1984)**  ***Aims:*** Evaluation of programme addressing cost-effectiveness of ordering diagnostic tests.  ***Setting:*** US, hospital based, 2^nd^ & 3^rd^ year medical students (64 intervention and 55 control). Simulated exercise -kidney stone.  ***Design:*** Mixed methods. Quantitative: pre/post intervention questionnaire survey covering knowledge about, and willingness to order, diagnostic tests and attitudes towards cost containment. Qualitative: open questions for control group only evaluating educational intervention. | | | |
| --- | --- | --- | --- |
|  | **Outcome measures** | | **Results** |
| **ALM** | Change in mean score on 7 items addressing cost-containment pre and post intervention. | | No significant difference between test scores. |
| **Wilkes et. al. (1994)a** (Health care reform as perceived by first year medical students) **and (1994)b** (Entering first-year medical students' attitudes toward managed care)  ***Aims:*** To examine medical students’ knowledge of current concepts in health services and attitudes toward changes taking place in the US health care sector.  ***Setting:*** US, 594 first year medical students entering 5 medical schools in the University of California System.  ***Design***: Quantitative: 139 item questionnaire assessing familiarity with health service. | | | |
|  | **Outcome measures** | | **Results** |
| **ALM** | Students’ attitudes towards: cost reduction; changes in US Health care system, and managed care | | Cost reduction: 72% felt physicians had responsibility to help reduce health care costs. 77% endorsed reducing health insurance company profits, 27% would reduce physicians’ salaries.  Changes in US healthcare system: 67% felt changes in the health care systems impaired physicans’ independence. 64% felt demands of physicians’ work interfere too much with family relations.  Managed care: male students interested in prestige and opposed to healthcare rationing were more likely to have negative attitudes towards managed care. Students interested in improving access to care and primary care careers more likely to have positive attitudes towards managed care. |
| **Lazarus et. al. (1998)**  ***Aims****:* a] to assess the level of knowledge about and attitudes towards managed care held by medical students, residents, faculty physicians and administrators b] to evaluate the impact of a 2 day course on knowledge and attitudes.  ***Setting:***US, 54 medical students (study also included faculty, administrators, and trainee doctors).  ***Design:*** Quantitative. Questionnaire measuring objective knowledge, self rated knowledge, attitudes and behavioural intentions at 3 time points: pre intervention, immediately post intervention and (shortened version) six months post intervention. | | | |
|  | **Outcome measures** | | **Results** |
| **ALM** | Change in mean scores on 4 multi-item attitudes scales. | | Attitude scales (mean score, max 5):  Influence of managed care on clinical quality pre-intervention mean 2.5, immediately post intervention +0.13, 6 months post intervention +0.19  Cost control effectiveness of managed care pre-intervention mean 3.3, immediately post intervention +0.62, 6 months post intervention +0.59  Need to adapt to managed care pre-intervention mean 3.6, immediately post intervention +0.13, 6 months post intervention +0.20  Perceived inevitability of managed care pre-intervention mean 3.5, immediately post intervention +0.25, 6 months post intervention +0.26 |
| **O’Connell et. al. (2004)**  ***Aims:*** To examine medical students’ attitudes towards Managed Care Organisations’ systems of care, and the effects of a teaching programme on these.  ***Setting****:* US, 658 medical students over 4 year programme, including 1 day visit to an Managed care organisation  ***Design:*** Quantitative. Both longitudinal and pre/post intervention study. | | | |
|  | **Outcome measures** | | **Results** |
| **ALM** | Changes in mean scores over period of programme (long term assessment).  Changes in mean scores before and after visit to MCO (short term assessment). | | No significant changes in mean scores on any of 14 items over 3 year period of programme before a day-long seminar at a nonprofit managed care organization. Significant positive changes in attitude found in pre and post visit scores for 8 of 14 items. |
| **Wilkes et. al. (1998**)  ***Aims:*** a] to measure the values and attitudes of medical students on entry to medical school, in comparison with two other professional students groups (business and law) b] to understand attitudes medical students brought to medical school  ***Setting:*** US, 120 medical 147 business and 173 law students attending 3 graduate schools of one university  ***Design:*** Quantitative. Questionnaire survey on course entry | | | |
|  | **Outcome measures** | **Results** | |
| **ALM** | Percentage of medical students agreeing with statements addressing attitudes regarding different areas of interest.  Areas where medical students differed significantly from other professional students. | >50% of medical students held the following views:  Managed care: that patients preferred fee-for-service, that Health Maintenance Organizations interfered with doctor/patient relationship, and Health Maintenance Organizations more concerned with economics.  Cost controls: that reforming medical malpractice system, increasing preventative health measures, price controls on doctors’ fees and decreasing compensation for specialists would be effective measures to reduce medical costs.  Allocation of resources: that people with bad lifestyles should pay more, that it was appropriate to withhold care on the basis of prognosis and there was a need to ration expensive medical technology.  Regulation of care by government: that government should be involved only in providing care to the poor, regulating the drug industry and motivating students into specialties.  Areas of difference:  Managed care: medical students more likely to feel that HMOs likely to interfere with MD-patient relationship, that HMOs more concerned with economics and that patients preferred private MDs.  Cost controls: medical students overall less in favour of cost control measures.  Allocation of resources: medical students less likely to endorse giving diagnostic tests on demand and withhold care on the basis of cost.  Regulation of care by government : medical students less likely to endorse role of government in defining medical school curriculum.  Access to care: medical students more in favour of routine preventive visits but less in favour of universal access for liver, and kidney transplant, coronary bypass and cataract surgery. | |
| **Mazor et. al. (2002)**  ***Aims:*** To examine graduating medical students’ perceptions of the adequacy of instruction in managed care and in 12 curricular content areas identified by experts as a necessary part of managed care education.  ***Setting:*** US 12,734 Graduating medical students.  ***Design:*** Quantitative. Questionnaire survey using Association of American Medical Colleges: national Medical School Graduation Questionnaire. | | | |
|  | **Outcome measures** | | **Results** |
| **AET** | Percentage students rating instruction in curriculum areas related to management as inadequate compared to other curricular content areas. | | Curricular areas: *Management areas: 41-72%*- [Cost control (57%) Cost effective practice (56%) Quality assurance (56%) Practice management (72%) Managed care (60%) Risk management (41%)].  Compared with *Practice management areas: 56-72%*; *Clinical decision making and clinical care areas: 9-21%; Population-based medicine areas: 20-40%* |
| **Noritz (1997)**  ***Aims:*** To investigate a] opinions of medical students towards managed care, b] exposure to managed care and c] perceptions of how managed care is addressed during medical education.  ***Setting****:* US, 53 medical students across all years in one medical school.  ***Design:*** Mixed methods. Semi structured interviews comprising 9 closed and 1 open question. | | | |
|  | **Outcome measures** | | **Results** |
| **AET** | Students’ opinions towards managed care  Students’ levels of exposure to managed care | | Students’ own opinions: 12% “Favourable” 75% “Mixed”. Students’ perception of instructors’ opinions: 2% “Favourable” 61% “Mixed”.  Level of exposure to: Managed Care Patients 8% “Often” 37% “Sometimes”; Managed Care Physicians 11% “Often” 38% “Sometimes”; Managed Care Discussions 19% “Often” 32% “Sometimes”.  Consideration of cost in clinical setting (n=32 3^rd^ & 4^th^ Year only) 47% “Often” 44% “Sometimes”. |
| **McEvoy & O’Brien (1985)**  ***Aims:*** To describe a resource allocation problem project undertaken by students and their evaluation of this.  ***Setting****:* UK, 50 2^nd^ year (preclinical) medical students at one medical school.  ***Design:* Mixed methods.**Questionnaire survey comprising closed and open questions evaluating project in terms of relevance to future careers. | | | |
|  | **Outcome measures** | | **Results** |
| **AET** | Responses to questions evaluating the project.  Free text comments.  Authors’ evaluations of students’ awareness and grasp of components of resource allocation problems. | | Responses: 45/50 students rated project as “very relevant” or “relevant” 48/50 rated project as “very interesting” or “interesting” and 45/49 rated talks and visits as “very useful” or “useful”.  Free text: 8/50 suggested more time be given in the undergraduate curriculum to exploring problems of the organisation and delivery of health care and allocation of limited resources.  Authors considered students displayed a] good grasp of financial constraints on the ambulance service and the need for efficiency and b] good understanding of patient expectations and practicalities of delivering the service. |
| **Agrawal et. al. (2005)**  ***Aims:*** To assess medical students’ perceptions about education at medical school in health care policy and delivery  ***Setting:*** US, 295/516 1^st^ year and 475/847 4^th^ year medical students throughout the US  ***Design:*** Quantitative. Questionnaire assessing knowledge of financing, performance and access to health care in US and importance and quality of learning experiences in health policy. | | | |
|  | **Outcome measures** | | **Results** |
| **AET** | Percentage of students agreeing or disagreeing with statements reflecting importance and quality of health policy education. | | 96% felt understanding health policy was important to practicing medicine. Approximately half expressed dissatisfaction with coursework related to health policy, delivery and reform issues and 89% wanted increased exposure to these issues. 67% of 4^th^ year and 48% of 1^st^ year students wanted increased medical school coursework. 54% of 4^th^ year and 60% of 1^st^ year students wanted increased elective medical school coursework. |
| **Tibbitts (1996)**  ***Aims:*** To report on graduating medical students perception of adequacy of training as indicated by  ***Setting:*** US, >12,000 students graduating from non-specified US medical schools.  ***Design:*** National survey (1993/4 Association of American Medical Colleges Medical School Graduation Questionnaire) evaluating 41 areas of medical school instruction. | | | |
|  | **Outcome measures** | | **Results** |
| **AET** | Areas of instruction where at least 50% of students rated training inadequate: | | 5 areas relating to management in which training rated as inadequate by at 50 % of students: Practice management (77.5%), Utilization review and quality assurance (73.1%), Cost effective medical practice (62.1%), Medical care cost control (61.9%), Medical social economics (61%) |
| **Toker (2007)**  ***Aims****:* To evaluate medical students’ knowledge about costs of laboratory tests, imaging procedures and treatments. To examine medical students' attitudes regarding receiving information about costs, and its effect on their future clinical behaviour.  ***Setting:*** Israel, 389 1^st^- 6^th^ year medical students.  ***Design:*** Quantitative. Survey Questionnaire. | | | |
|  | **Outcome measures** | | **Results** |
| **AET** | Percentage of respondents who remembered if given any formal knowledge about medical costs.  Percentage of respondents who were interested in acquiring information regarding medical costs.  Percentage of respondents who did think there is a need to include economic material in the medical school curriculum.  Percentage of respondents who did think that knowledge about medical costs will influence their clinical decisions in the future. | | 70.8% did not receive any information about medical costs. 21.6%/7.6% did /did not remember if any information given.  83.9% replied they would like to acquire information regarding medical costs. 16.1% replied they would not be interested.  65.4% did think there is a need to include economic material in the medical school curriculum. 34.6% replied there is no such need.  69% did/ 31% did not think that knowledge about medical costs will influence their clinical decisions in the future. |

**Content Area: General leadership and management**

| **Varkey et. al. (2009)**  ***Aims:*** To elicit the perspectives of students, faculty physicians and administrators regarding knowledge and competences necessary in an undergraduate leadership curriculum.  ***Setting:*** US, 21 elected medical student leaders drawn from all years.  ***Design:*** Mixed methods. Questionnaire survey of purposeful sample of students covering perceived importance and self rated competence in 13 areas of leadership, and views on inclusion of leadership in curriculum. | | |
| --- | --- | --- |
|  | **Outcome measures** | **Results** |
| **ALM** | Student ratings of the importance of 13 different areas of leadership | Leadership skills regarded as “very important” by:  >85% of students: Communication skills, Ethics, Conflict Resolution, Time Management.  50% -70% of students: Managed care, Management principles, Coding and billing, Quality improvement, Public speaking, Risk management.  < 50% of students: Negotiating , Writing proposals , Investment |
| **AET** | Proportion of students favouring inclusion of leadership education in the curriculum . | 85% felt that leadership, communication, teamwork and quality improvement skills should be taught in medical school, with remaining (15%) stating ‘maybe’. |
| **Carufel-Wert (2007)**  ***Aims:*** To report the effects of the LOCUS [Leadership Opportunities with Communities, the Underserved, and Special populations] programme on participants and their evaluation of it.  ***Setting:*** US, 50 students (ranging from 1^st^ to final year) previously or currently involved in programme.  ***Design:*** Mixed methods. Questionnaire survey covering students’ background, reasons for enrolling, programme experience and evaluation. | | |
|  | **Outcome measures** | **Results** |
| **ALM** | Student assessment of impact of programme on attitudes towards leadership positions. | Positive impact score 1.14 (max 2.0) on ‘Interest in taking leadership positions’. |
| **AET** | Percentage giving leadership training as reason for enrolling in programme.  Percentage giving positive evaluation of programme. | Leadership training reason for enrolling: 96% (however rated as 5^th^ most important out of 7 items).  Positive evaluation: 62% ‘definitely’ 22% ‘generally’ considered programme worthwhile, but 10% described negative impacts of programme on coursework, 8% on ability to juggle competing demands. |
| **Whittle & Murdoch Eaton (2001)**  ***Aims:*** To assess attitudes of first year undergraduates towards transferable skills, gender differences in these, and impact of course on skill development.  ***Setting****:* UK, 109 First year medical students.  ***Design:*** Quantitative. Questionnaire survey at the end of the first year addressing: perceived importance, self assessed ability and influence of first year course on a number of transferable skills including on organizational and self learning skills. | | |
|  | **Outcome measures** | **Results** |
| **ALM** | Mean score (range 1-4) for perceived importance of skills. | Most skill areas rated highly (mean>3.0). Generally perceived importance scored more highly than their self-rated competence. |
| **Martins et. al. (2005)**  ***Aims:*** To explore and compare the opinions of UK and Portuguese medical students on the value and structure of a ‘stand-alone’ management and leadership course.  ***Setting:*** UK & Portugal 1^st^ year medical students at University of Cambridge (n= 141) and University of Lisbon (127)  ***Design:*** Mixed methods. Semi-structured questionnaire survey of medical students and in-depth interviews with hospital manager and clinical director at each site. | | |
|  | **Outcome measures** | **Results** |
| **AET** | Percentage distribution scores evaluating relevance (rated 1-4 ).  Differences in opinions between students who rated leadership/management education highly relevant and students whose rating was lower.  Percentage distributions regarding students views regarding the time, nature and length of a a potential course.  Percentage distribution of ranking of importance (determined as students’ ranking 1st-4^th^ most important area) seen for 12 specified possible course content areas | Relevance: Leadership and management course considered of high relevance. Score of 3 or 4 given by students in Cambridge 42% and Lisbon 63%. Difference statistically significant p<0.001.  High relevance group mentioned improvement of self-confidence, increase NHS efficiency/effectiveness, necessary role of doctors, ability to assume further responsibilities. Low relevance group mentioned curriculum pressures, doctors should only be concerned with patients.  Student views regarding potential course (only preferred options reported here): during clinical years: 79% Cambridge, 61% Lisbon; optional 58% Cambridge, 62% Lisbon; 1 term/semester assuming 4 hours per week and a respective reduction in other subjects’ teaching time 56% Cambridge, 73% Lisbon.  Course content areas ranking 1-4 (percentage of students):  a] “Managing people” seen as most important by both sets of students (approx 20%).  b] Cambridge “Doctor and Leadership” (18%) followed by “ Costs and pricing” (8%) and “Management of learning personal development” (8%).  c] Lisbon “National Health System” (15%), followed by “Management aspects of Public Health” (12%) and “Clinics and hospitals as complex organisations” (12%). |

**Content Area: Role of Doctor**

| **Harward (2006)**  ***Aims:*** To explore whether participation in an interdisciplinary case conference changed medical students’ knowledge and attitudes about the role of interdisciplinary teams in health care.  ***Setting:*** US, 605 2^nd^ year medical students (over 4 years).  ***Design:*** Quantitative. Pre/ post intervention questionnaire survey | | |
| --- | --- | --- |
|  | **Outcome measures** | **Results** |
| **ALM** | Level and changes in mean scores for attitudes towards interdisciplinary teams: (max 5).  Level and changes in mean scores for attitudes towards leadership of interdisciplinary teams by other health professionals: (max 5).  Level and changes in mean scores for perception of importance of care provided by other health professionals: (max 5).  Level and changes in mean scores for perceived importance of activities relating to the functioning of interdisciplinary teams: (max 5). | Pre intervention: positive attitudes on 3 of 4 items: team improves a]care quality, b] likely to results in patient treated as whole person and c] fosters enthusiasm and interest (range 3.55 to 4.31)  Post intervention statistically significant increases on all of these (range +0.13 to 0.32)  Pre intervention: Mixed views on 1 item “teams complicate things”. No change post intervention  Pre intervention students held relatively low attitudes towards leadership of teams by health professionals other than doctors, or nurses  Minimal (</=2.5) audiologist, Low (>2.5</=3.0) dentist, occupational therapist, pharmacist, physical therapist, social worker, speech pathologist, rehabilitation counsellor.  Post intervention small statistically significant improvements in all except audiologist (range +0.09 to +0.33)  Pre intervention students held relatively high positive views of importance of care provided by other health professionals (mean score >4.00)  Exceptions: audiologists (2.72), speech pathologist (3.93), rehabilitation counsellor (3.69)  Post intervention significant improvement only in audiologist (+0.67) and pharmacist (+0.22), others small significant decline (<0.21) or no change  Pre intervention students held relatively high positive views of importance of a] “collaborative care planning” b] “understanding others’ terminology” and c] “negotiating treatment and follow up” (mean score >4.00) Slightly less positive views about importance of “meeting face to face” (3.92)  Post intervention small but statistically significant improvements only in “meeting face to face” and “negotiating treatment and follow up” (+0.07) |
| **Holmstrom et. al. (2004)**  ***Aims:*** To determine students’ thoughts regarding their professional role in future health care.  ***Setting:*** Sweden, 57, 4^th^ year students.  ***Design:*** Qualitative assessment of student essays. | | |
|  | **Outcome measures** | **Results** |
| **ALM** | Main themes identified from 2 page essays giving views on professional role in future health care. | 1] Professional role changing: need for greater clarity in leadership role, students rejected hierarchical organization but at same time felt that doctors should be leaders. Rejected paternalistic role vis a vis patients. Saw need to promote co-operation and communication between health care professionals.  2] Regarded current system (Swedish) as unsatisfactory from both patient and health care professional. Saw greater use of private alternatives and competition but wary of adopting US approach.  3] Concern about burnout and escalating demands. Public sector not seen as attractive as private sector. |
| **Cavenagh (2002)**  ***Aims:*** To investigate the effects of new curriculum on medical students’ attitudes and beliefs towards the roles of the doctor: in particular management role  To ascertain medical students’ interest in concept of doctors in NHS trust management  ***Setting:*** UK, 1995-2000, 2 medical schools differentiated by approach: traditional/modern PBL. At each medical school: First and final year medical students from “old” and “new” curriculum  ***Design:*** Quantitative. Questionnaire survey administered to the four cohorts on two occasions: entry to and exit from course. Self rated knowledge of role of clinical director assessed using 7 point scale for final year students only. Questionnaire piloted. | | |
|  | **Outcome measures** | **Results** |
| **ALM** | Mean attitude score (max 5) and comparisons between students over time (longitudinal comparison), between medical schools (cross-sectional comparison).  Attitudes of students exiting courses at each medical school. | **Longitudinal comparisons:**  **(Repeaters/same individuals)**  Medical School A no change in students’ attitudes towards management education overtime on either curriculum (Range 3.0 – 3.5 out of max of 5)  Medical School B no change in students’ attitudes towards management education overtime on old curriculum but significant reduction in interest in management education overtime on new curriculum.  (Range 3.1 – 3.7 out of max of 5)  **Cross sectional comparisons**  Medical school A students entering the new curriculum had significantly more positive views towards management education than those entering old curriculum.  No differences found at either entry or exit for when comparing old and new curricula at medical school B  **Final year students’ attitudes**  In Medical School A students who exit new curriculum displayed more positive attitudes towards management education (but still “undecided”) than those who exited old curriculum:  Medical School B no differences were found in final year students’ attitudes towards management education between those exiting new curriculum and those exiting old curriculum. |

**Content Area: Patient Safety**

| **Madigosky (2006)**  ***Aims:*** To investigate of the effects of a patient safety and medical fallibility curriculum on students’ knowledge, skills and attitudes immediately following an educational intervention and one year later.  ***Setting****:* US, hospital based, 92, 2^nd^ year medical students.  ***Design****:* Mixed qualitative and quantitative, pre/post intervention and longitudinal study. Questionnaire survey including open comments administered pre-intervention, immediately post intervention and 1 year later. At one year posttest students asked to report behaviours | | |
| --- | --- | --- |
|  | **Outcome measures** | **Results** |
| **ALM** | Direction of change in mean scores for 16 attitudinal items between 3 time points. | Student attitudes improved immediately after intervention in 5 items: “Errors inevitable, Need to work harder, Competent doctors don’t make errors, Doctors share information”. After one year, improvement was sustained in all of above but some deterioration in attitudes towards “Usefulness of reporting systems”  Student attitudes became more unfavourable immediately after intervention in 5 items: “Doctors should work to improve patient care, Easy for providers to deal with errors, Patient safety appropriate use of time in med school, If I saw error would keep it to self, If no harm to patient no need to address”. After one year there was no alteration in direction of attitude change and some “hardening” of negative attitudes.  Student attitudes remained unchanged both immediately after the intervention and one year later for 6 items: “Gap between best care and what we provide, Errors due to things outside doctors’ control, Only doctors can determine causes of error, Faculty & staff communicate importance of patient safety, Doctors should not tolerate uncertainty in patient care”. |
| **AET** | Proportion of students giving positive evaluation to intervention.  Content analysis of qualitative responses. | Positive student evaluation: “Meeting learning objectives” 72%, “Usefulness in medical education” 73%, “Of benefit to career” 82%, “Support for continuing” 72%  Content analysis: Most important gains from course: Everyone makes mistakes. Methods of addressing root causes of errors. Error reporting and disclosure important/ |
| **Goldie et. al. (2003)**  ***Aims****:* Examination of students’ attitudes towards, and potential behaviour in respect of, whistle blowing.  ***Setting****:* UK (Scotland) Longitudinal cohort following 162 incoming (Year 1) medical students  ***Design:*** Mixed methods. Longitudinal cohort design. The adapted Ethics in Health Care Survey Instrument (EHCI) used at 4 time points: pre-year 1, post- Year 1, post Year 3 post year 5. Paper reports findings for 1 ethically challenging vignette concerned with whistle blowing. | | |
|  | **Outcome measures** | **Results** |
| **ALM** | Proportion of students giving answers reflecting the prevailing professional consensus to multichoice questions.  Proportion of students providing written justifications for their answers which reflect prevailing professional consensus.  Content analysis of students’ written justifications for their answers. | Pre year 1, 56% (n=50), post Year 1, 75% (n=40), post year 3, 60% (n=30) and post year 5, 62% (n=50).  Pre year 1, 36% (n=50), post Year 1, 40% (n=40), post year 3, 37% (n=30) and post year 5, 30% (n=50).  Little improvement in students’ performance in terms of hierarchies of justification and hierarchy of values (tending to reflect student’s values/morality)  “Not my place to criticize” one of top 4 reasons for not whistle blowing at each time point. |
| **Halbach et. Al. (2005)**  ***Aims****:* To assess the effectiveness of a brief curriculum about patient safety and medical errors with third-year medical students.  ***Setting****:* US 572 Year 3 medical students over 3 academic years.  ***Design:*** Quantitative. Pre and post intervention questionnaire comprising 7 self evaluation items (5 point Likert scale) addressing self-awareness regarding aspects of patient safety and 13 course evaluation items. 2-8 month follow up 12-item questionnaire survey. No control group. | | |
|  | **Outcome measures** | **Results** |
| **AET** | Percentage agreement with statements regarding course evaluation           Percentage agreement with statements on follow up questionnaire | 89% of students agreed that ‘the opportunity to present an error to a patient increase my confidence about discussing this issue with patients’.  58% agreed that ‘the readings were helpful’  94% of students reported the standardized patient exercise to be ‘a valuable learning experience’    In follow up 97% agreed that ‘it is important to teach students about medical errors’;  87% agreed that ‘the third year is the more appropriate time to discuss medical errors.’ |
